# Feasibility and acceptability of a personalised script-elicitation method for improving evening sleep hygiene habits

**DOI:** 10.1080/21642850.2022.2162904

**Published:** 2023-01-01

**Authors:** Anya Mohideen, Clara Bouvin, Gaby Judah, Federica Picariello, Benjamin Gardner

**Affiliations:** a Department of Psychology, King's College London, London, UK; b Patient Safety Translational Research Centre, Imperial College London, London, UK; c Department of Psychology, University of Surrey, Guildford, UK

**Keywords:** Sleep, behaviour change, habit, trial, mixed methods

## Abstract

**Background:**

Attempts to improve evening sleep hygiene have overlooked that sleep preparation behaviours are often undertaken automatically with little awareness; that is, habitually. This mixed-methods study assessed aspects of the feasibility and acceptability of a novel behavioural intervention procedure (‘script elicitation’), which encourages reflection on and reorganisation of the content and sequencing of habitual evening pre-sleep routines.

**Methods:**

The study was advertised via social media, and circular lists at a UK university. Twenty-four UK-based adults, reporting <6 h/night sleep, were recruited. At baseline, they completed sleep hygiene and quality measures, then participated in an online, one-to-one script elicitation interview. This involved the interviewer working with the participant to generate a fine-grained description of the content, organisation and variability of their typical pre-sleep routine, and plan a more sleep-conducive alternative routine to follow over the next week. One week later, participants completed sleep quality and hygiene measures, and a semi-structured interview about the intervention. Feasibility was assessed using quantitative data on response rates and attrition, and acceptability via sleep hygiene and quality scores, and qualitative data on intervention experiences.

**Results:**

All criteria were met. The target response rate was exceeded, none of the 24 participants dropped out, and sleep hygiene and quality scores either improved or remained stable. In interviews, all participants reported finding script elicitation useful. Script elicitation raised participants’ awareness of habitual sleep hygiene routines, which gave many a newfound sense of autonomy over changing their sleep hygiene habits. While the habitual nature of existing routines obstructed change for some participants, most reported successfully changing aspects of their routine, and achieving behaviour, sleep and wellbeing improvements.

**Discussion:**

Script elicitation is a promising and acceptable method for tackling poor evening sleep hygiene habits. A more rigorous trial is warranted.

## Introduction

A third of UK adults fail to achieve the six hours of daily sleep recommended for optimum health (Consensus Conference Panel et al., 2015; YouGov, 2022), and a quarter report sleeping ‘poorly’ or ‘very poorly’ (Sleep Council, 2017). Not getting enough good quality sleep is linked to physical health problems, including cardiovascular disease, type 2 diabetes and obesity (Itani et al., 2017; Watanabe et al., 2010). Poor sleep hygiene – i.e. engaging in sleep-inhibiting behaviours, such as consuming caffeine late in the day, or working or using electronic devices within 30mins of attempting to sleep (Sleep Foundation, 2022) – disturbs sleep (Peltz & Rogge, 2016). Poor evening sleep hygiene is common: for example, 65% of UK adults report using social media in bed (The Sleep Council, 2017). This is problematic because not only does engaging with social media maintain cognitive arousal and disturbs sleep (Levenson et al., 2016), but also, in the UK, social media is most commonly accessed via phones (We Are Social, 2022), blue light emissions from which adversely affect sleep (Oh et al., 2015). Effective sleep hygiene behaviour change interventions are needed.

Behavioural sleep hygiene interventions have typically generated only small effects on sleep duration or quality (Murawski et al., 2018). This may be due to misplaced assumptions that poor sleep preparation represents intentional neglect of sleep (e.g. Mead & Irish, 2022). Sleep hygiene interventions have typically involved motivational techniques, such as informing people of the consequences of unhygienic sleep preparation (Blunden et al., 2012). Yet, people generally recognise the importance of sleep for health, suggesting lack of knowledge or motivation is not the cause of poor sleep hygiene (Paterson et al., 2019).

Unhygienic evening sleep hygiene behaviours are, for many people, likely to be executed habitually (Rebar et al., 2020). Habitual actions are triggered automatically when people encounter situations in which they have previously acted, due to the activation of learned situation-action associations (Gardner, 2015). Due to their automaticity, habitual behaviours proceed rapidly and efficiently, without requiring conscious awareness, control, or motivation (Bargh, 1994). People can act habitually without intention, and even when they intend to perform non-habitual alternatives (Gardner et al., 2020a). Unwanted habits can thereby undermine sleep hygiene motivation. One study showed that young adults’ evening sleep hygiene enhancement attempts were commonly derailed by ‘bad’ habits such as eating before bed (Paterson et al., 2019). Interventions should seek to displace unhygienic habits with sleep-conducive alternatives (Rebar et al., 2020).

Breaking bad habits requires different techniques to those needed to change conscious motivation (Gardner et al., 2020b). People should be encouraged to adopt hygienic alternatives when they encounter cues to unwanted habitual behaviours. Yet, because habitual behaviours are prompted automatically, people often lack insight into cues, their responses to them, or the relationship between cue exposure and their response (Hollands et al., 2016). ‘Script elicitation’ is a novel, theory-based method to identify and modify habitual responses (Judah et al., 2013). As administered via a one-to-one interview, script elicitation involves an interviewer supporting the participant to describe in fine detail the sequence (or ‘script’) of actions (e.g. ‘brush teeth’) and cues within a given behavioural episode (e.g. ‘getting ready for bed’). This process may change behaviour through two mechanisms. First, it may raise conscious awareness of cues (e.g. preceding behaviours, events, or locations), responses and cue-response links. It encourages conversion of procedural knowledge regarding *how* sleep preparation sequences are undertaken, into declarative knowledge *of* specific acts and cues within the sequence (Anderson, 1983). Second, elicited scripts provide a basis for reorganising, removing, curtailing, or adding behaviours within the sequence. For example, an oral health intervention used script elicitation to identify the optimal position at which to place flossing within personalised routines (Judah et al., 2013).

### The present study

Many common behaviours that precede sleep, such as using electronic devices, are controlled by habit rather than conscious motivation (Exelmans & Van den Bulck, 2021). Script elicitation is an intervention procedure rooted in habit theory (e.g. Gardner et al., 2016), and involves participants articulating the content and organisation of habitually executed routines as a means of raising conscious awareness of those routines. Script elicitation has shown promise for changing oral health and handwashing (Greenland et al., 2013; Judah et al., 2013), but has not been applied to sleep hygiene.

Medical Research Council guidance describes four phases in intervention development and evaluation: development or identification of the intervention, feasibility assessment, evaluation, and implementation (Skivington et al., 2021). The present study, which coincided with the first national Covid lockdown in the UK, was situated within the second of these phases. The study was designed to assess the feasibility and acceptability of script elicitation for promoting evening sleep hygiene, to inform a decision on whether to progress to a controlled trial. Feasibility – i.e. the extent to which our intervention could be implemented – and acceptability – i.e. whether recipients are willing to receive and adhere to the intervention (Lancaster et al., 2004) – are essential for intervention effectiveness. Feasibility and acceptability studies establish whether devoting resources to more rigorous and expensive effectiveness trials is justified (Skivington et al., 2021). We used quantitative data to capture two feasibility indicators (response rate, attrition), and one acceptability (no detriment in sleep hygiene or quality). Qualitative data explored acceptability more freely.

## Participants and methods

### Participants, design and procedure

A single-arm, pre–post mixed-methods design was used. Participants were recruited in June 2020 using convenience sampling methods, via online advertisements on social media (LinkedIn, Facebook, Twitter) and an advert in an all-staff circular email at an inner-city UK university. Adverts described the study as an opportunity to trial a new sleep improvement method and directed potential participants to an online survey through which they self-declared eligibility. Inclusion criteria were: aged ≥18y; UK resident; and typically sleeping <6 h per night. Exclusion criteria were: referred for clinical treatment for insomnia; short sleep attributable to medication, illness, or shift work; caring responsibilities for children under 7y; recent acute illness or operation; or known to researchers. Ineligible participants were unable to finish the survey. Eligible participants next provided informed consent, self-reported demographics (Table 1), typical sleep duration and quality, and sleep hygiene behaviours. They provided their interview availability, and email address to receive interview confirmation. Aside from email correspondence to arrange the interview, no relationship was established with any participant prior to the interview, nor did participants have any knowledge about the interviewer other than that they were a psychology researcher who wished to test a method for improving sleep hygiene.

**Table 1.** Sample characteristics (*N* = 24).

		*N*	%
*Typical sleep, weekdays*	Mean 5.4 h, range 4–6 h	–	–
*Typical sleep, weekend days*	Mean 5.5 h, range 4–6 h	–	–
*Age*	Mean 29y, range 18–66y	–	–
*Gender*	Female	19	79%
	Male	5	21%
*BMI*	Mean 24.31, range 17–39	–	–
*Ethnicity*	White	17	71%
	Asian/Asian British	5	21%
	Mixed	1	4%
	Not listed	1	4%
*Occupation*	(Full-time) student	13	54%
	(Full-time) employed	8	33%
	Furloughed	1	4%
	Retired	2	8%
*Education*	O Levels, GCSEs, foundational diploma	2	8%
	A Levels	4	17%
	Undergraduate	11	46%
	Postgraduate	7	29%
*Marital status*	Single	16	67%
	Married	5	21%
	Cohabiting	3	13%
*Living arrangement*	Living with parents	10	42%
	Living with partner	9	38%
	Living with partner and child	1	4%
	Living with non-relatives	3	13%
	Living alone	1	4%

Participants next completed an online interview via Microsoft Teams involving: pre-intervention questions about sleep; the script elicitation procedure; and immediate post-intervention reflections. Script elicitation generated both a personalised current script, and a co-created alternative script intended to guide behaviour over the coming week, a flow chart of which we emailed to participants immediately post-interview. Only the interviewer and participant were present in each interview. Interviews were undertaken by one of two female postgraduate (Masters) Health Psychology students (AM, CB). Neither had conducted real-world research interviews previously. Both were trained in research interviewing by BG and script elicitation administration by GJ. These interviews lasted between 17 and 59 mins (mean 36 min, *SD *= 10).

One week later, participants received a second online survey link, via which they reported sleep hygiene and quality. All participants responded, so no reminders were required. They also gave a second, semi-structured interview regarding their experiences over the preceding week. The second interviews lasted between 2 and 23 mins (mean 8 min, *SD *= 4). Participants who completed both interviews received a £20 voucher. Both interview schedules were piloted by AM and CB in mock interviews with friends.

Both interviews were audio-recorded, and recordings of interviews were transcribed verbatim using automated software (Otter.ai; Corrente & Bourgeault, 2022). Transcripts were manually checked for accuracy (by AM and CB) against interview recordings. All procedures were approved by the King’s College London Ethics Committee (LRS-19/20-18414).

The project was undertaken as Masters coursework, budgetary limits for which imposed a maximum sample of 24 participants. Participants entered the study on a first-served basis. Those who came forward after 24 participants had signed up were added to a reserve list, but nobody dropped out, so the reserve list was not used. Twenty-four participants completed the study (19 females, 5 males; age 18–66 years [Mean = 29.3y, *SD = *13.2y]). Participants reported sleeping on average 5.4 h/night on weekdays, and 5.5 h at weekends. They were most commonly White British (58%), full-time students (54%), and living with their parents (42%; see Table 1).

The study coincided with the Covid pandemic, which is why data collection was run online. The UK was in national lockdown throughout recruitment. Twenty of 24 baseline interviews, and nine follow-up interviews, were conducted during lockdown. Four baseline interviews were run after lockdown was lifted, and for eleven participants, lockdown ended between baseline and follow-up interviews. We have no reason to expect that lockdown restrictions unduly affected intervention experiences.

### Script elicitation intervention

Script elicitation procedures were administered at the baseline interview by AM or CB. The aim was to identify the content and sequencing of participants’ personalised evening sleep hygiene routines, and to support them to design a sleep-conducive alternative script. As participants spoke, the interviewer noted and visually organised responses on a Microsoft Word document, visible to participants via screen-sharing. A description of the behaviour change techniques underlying use of script elicitation to identify existing scripts and design new scripts respectively is provided as Supplementary Material (see Michie et al., 2013).

*Eliciting current sleep hygiene script.* Participants were first asked to describe their typical evening routine from ‘getting ready for bed’ to ‘falling asleep’. Next, they identified which listed activities ‘went together’. This enabled clustering of behaviours (e.g. ‘check social media’, ‘use FaceTime’) into higher-order units (e.g. ‘use my phone’), and separation and sequencing of higher-order units (e.g. ‘finish working’, ‘use my phone’). Participants next described the first and last event within each activity, to identify potential cues. This process continued until interviewer and participant agreed that written and visual script depictions were accurate, comprehensive and specific.

*Co-creating an alternative script for behaviour change.* Next, participants collaborated with the interviewer to develop an alternative version of their script, to which they would attempt to adhere over the coming week. The current script was copied into a new, screen-shared Microsoft Word document, edited as the participant spoke. Participants were invited to use one or more of three strategies: add new behaviours, remove or curtail existing behaviours, or re-organise existing behaviours. When participants wished to remove existing behaviours, they were encouraged to substitute them with specific, preferred alternatives, because simply omitting an automated action can heighten impulses to enact that action (Lally & Gardner, 2013). Participants were not given guidance on which behaviours to add to or remove from their routine, and while it was intended that the interviewer would intervene if participants added new behaviours that were ‘unhygienic’ according to recommendations (Centers for Disease Control and Prevention, 2016; NHS, n.d.), no participant did so. If participants struggled to adapt their routine, the interviewer offered suggestions consistent with sleep hygiene recommendations (Centers for Disease Control and Prevention, 2016; NHS, n.d.).

Participants were also asked to identify explicit cues, by specifying, for example, events, times, or locations to precede performance of new or reorganised behaviours. The interviewer codified each desired behaviour change into specific, personalised action plans (‘when I encounter cue X, I will do Y’) to aid adherence. This continued until interviewer and participant agreed the new script was a credible, sufficiently detailed plan to which participants could feasibly adhere over the coming week.

For illustrative purposes, behaviours that were reorganised, added or removed from each script were coded from interview transcripts by BG (see Table 2).^1^ Example of current and alternative scripts are presented in a Supplementary Figure.

**Table 2.** Summary of planned behaviour changes in alternative scripts (*N* = 23).

Behaviours reorganised within script *(placed earlier and/or curtailed)*	Behaviours added to script	Behaviours removed from script
− Misc phone/device use (*N* = 13) − Going to bed (*N* = 5) − Working (*N* = 5) − Eating evening meal (*N* = 3) − Making phone calls (*N* = 2) − Reading (*N* = 2) − Watching TV (*N* = 3) − Drinking soothing drink (*N* = 1) − Drying hair (*N* = 1) − Exercising (*N* = 1) − Taking dog out (*N* = 1) − Using social media (*N* = 1)	− Reading (*N* = 9) − Doing yoga (*N* = 5) − Showering (*N* = 2) − Writing lists (*N* = 2) − Breathing exercises (*N* = 1) − Contemplating (*N* = 1) − Drinking soothing drink (*N* = 1) − Exercising (*N* = 1) − Listening to podcast (*N* = 1) − Listening to relaxing sounds (*N* = 1) − Meditation (*N* = 1)	− Watching TV (*N* = 6) − Misc phone/device use (*N* = 4) − Working (*N* = 2) − Drinking hot chocolate (*N* = 1) − Having a bath (*N* = 1) − Waiting for others to go to bed (*N* = 1)

NB: Data are missing for one participant due to human error.

### Data collection

Study data are available at https://osf.io/7963j/?view_only=9ec3667882f84e1cae56d463453c6e06.

*Quantitative data.* Study *response rate* reflected the number of people who contacted the research team to express interest within a week of study advertisements being posted. *Attrition* was operationalised as a failure to complete the follow-up measures or interview.

*Sleep hygiene* was self-reported via the 13-item Sleep Hygiene Index (SHI; Mastin et al., 2006), which assesses common pre-sleep behaviours (e.g. ‘I do important work before bedtime’) on 0–4 Likert scales (*Never* [0] – *Always* [4]). Scores were reversed then summed (0–52), higher scores indicating better hygiene. Reliability was good (baseline α = .76; follow-up α = .81). *Sleep quality* was self-reported using the 10-item Leeds Sleep Evaluation Questionnaire (LSEQ; Parrott & Hindmarch, 1980), which comprises four domains. Three domains were tapped by multiple items with a common stem: *getting to sleep* (three items; e.g. ‘How would you describe the way you currently fall asleep in comparison to usual?’ [*Slower than usual* – *Quicker than usual*]); *quality of sleep* (two items; e.g. ‘How would you describe the quality of your sleep compared to your normal sleep?’ [*More restless than usual – Calmer than usual*]); and *awake following sleep* (two items; e.g. ‘How would you describe your awakening in comparison to usual?’ [*More difficult than usual – Easier than usual*]). A fourth domain – *behaviour following wakening* *–* was assessed via three items (e.g. ‘How do you feel when you wake up?’; [*Tired – alert*]). Each was measured on visual analogue scales (0–10). Higher summed values (0–100) reflected better sleep quality.

*Qualitative data.* The *baseline interview* comprised questions about existing sleep patterns, previous sleep improvement attempts and, after script elicitation, experiences of the intervention procedure. The *follow-up interview* probed experiences of attempting to adhere to the new script, and further reflections on script elicitation. Both interview schedules are provided as Supplementary Material.

### Progression criteria and analyses

Feasibility was assessed via two quantitative indicators (response rate, attrition). Acceptability was assessed using a quantitative indicator (no detriment in sleep hygiene and quality outcomes), and qualitative data capturing participants’ spontaneous reflections. We intended our analyses to capture four dimensions of acceptability: affective attitudes towards the intervention (e.g. positive reflections on the intervention), participant burden (e.g. willingness to adhere to new scripts), ethicality (i.e. no harm caused), and perceived effectiveness (Sekhon et al., 2017). Progression criteria were set, for quantitative indicators, to determine whether procession to a controlled trial was warranted.

*Feasibility progression criteria.* Study *response rate* was deemed adequate if ≥12 participants expressed interest within one week of the study advertisement. Following Murawski et al. (2018), who observed 16% omnibus drop-out from behavioural sleep health interventions in non-clinical adult samples, *attrition* was deemed adequate if no more than four participants (i.e. 16% of target sample) dropped out between baseline and follow-up.

*Acceptability criteria.* Observed *patterns of change in sleep hygiene and quality* were deemed acceptable if there was no apparent worsening between baseline and follow-up in SHI total scores, any LSEQ domain, or summed-total LSEQ. Qualitative data were exploratory, so no criteria were set.

*Quantitative analyses.* Quantitative data were analysed descriptively. Although not used to formally assess patterns of changes in sleep outcomes due to lack of power, non-parametric Wilcoxon signed-rank tests were run for illustrative purposes. Non-parametric tests, based on median values, were used due to skewed baseline and follow-up values.

*Qualitative analyses.* Verbatim transcripts of post-script-elicitation data from baseline interviews, and follow-up interviews, were analysed using the ‘codebook’ form of inductive Thematic Analysis (Braun & Clarke, 2019, 2021). Analysis involved data familiarisation, coding, theme extraction, theme review, theme naming, and narrative analysis. Our ‘codebook’ methods involved multiple coders generating an initial coding framework, which guided later analysis. Specifically, AM, CB and BG first independently familiarised themselves with and subsequently coded three transcripts, assigning labels to pertinent events within the data. Next, the three coders met to agree a preliminary, inductively derived structure of codes and clusters of codes (i.e. themes), to guide subsequent analysis. Next, this structure was applied and iteratively refined by AM to code all remaining data. AM met regularly with BG to review themes, assign appropriate theme labels, and confirm credibility of interpretations. Analysis continued until AM and BG agreed that no meaningful additional themes or codes were emerging.

‘Codebook’ methods were used not only to ensure that preliminary interpretations from multiple coders could be drawn on throughout the analysis process, but also to allow BG, as the project supervisor, to use initial coding activities as a training opportunity for AM and CB, and to offer a coding guidance tool to AM (Braun & Clarke, 2021).

*Researcher positionality.* Three authors were involved in designing and executing data collection and analysis procedures. AM is an Asian British female, who was aged 22y and typically slept for 8 h per night during the study, and CB a White European female, aged 21y who also typically slept 7–8 h per night. BG is a White British male, aged 39y, who typically slept around 5–7 h per night, and often less than 6 h. All authors believe that sleep hygiene is an important determinant of sleep quality. AM and CB’s lack of personal experience of short sleep may have impacted their understanding of consistent short sleepers’ experiences. BG’s personal experience of short sleep is likely to have influenced his supervisory guidance and input on the qualitative interview schedules. None of the authors had previously undertaken empirical research in sleep hygiene, nor are they sleep specialists.

AM and CB’s primary motivation for undertaking the study was to satisfy postgraduate coursework requirements, though both had chosen this project from a predefined list of potential health psychology coursework projects. As an expert in habit theory and research and having been involved in previous script elicitation work (Judah et al., 2013), BG was motivated by a keenness to develop, assess, and obtain proof of concept for the script elicitation method as a means of understanding and changing habitually executed behaviour sequences (see Gardner et al., 2016).

## Results

### Quantitative data

*Response rate.* Within the first week of advertising the study, 96 potential participants contacted us to express interest, exceeding our threshold of 12 participants.

*Attrition rate.* All 24 participants completed follow-up (0% attrition), exceeding our 16% threshold.

*Sleep outcomes.* As Table 3 shows, sleep hygiene scores remained stable (*p* = .26). Scores on all sleep quality indices improved between baseline and follow-up and, while the study was underpowered to detect true effects, observed effects were sufficiently large to reach statistical significance (minimum *p* = .004). No deterioration was observed, so acceptability criteria were met.

**Table 3.** Sleep hygiene and quality at baseline and follow-up (*N* = 24).

		No. of items	Possible range	Baseline Mean (median, SD)	Follow-up Mean (median, SD)	*p* for median differences
*Sleep hygiene*		13	0–52	31.83 (32.50, 7.46)	33.63 (33.50, 7.47)	.26
*Sleep quality*	Getting to sleep	3	0–30	13.95 (15.10, 5.32)	18.48 (18.40, 3.65)	.004
	Quality of sleep	2	0–20	6.99 (5.95, 3.94)	11.95 (12.05, 4.59)	<.001
	Awake following sleep	2	0–20	7.23 (6.10, 4.85)	11.12 (11.50, 3.53)	.003
	Behaviour following wakening	3	0–30	11.07 (10.65, 4.41)	16.80 (16.15, 5.48)	<.001
	*Total*	10	0–100	39.24 (37.75, 12.79)	58.36 (59.95, 12.69)	<.001

### Qualitative data

Four themes were extracted. Two themes related to the script elicitation process (acceptable components of script elicitation; raised awareness of own sleeping patterns), and two to implementation and perceived benefits (barriers and facilitators of adherence to new scripts; experienced behavioural and health changes).

*Acceptable components of script elicitation.* All participants found the script elicitation procedure helpful. Many valued verbalising their existing routines and having them depicted in flow-charts. Some felt this allowed them to view their behaviour more objectively, which highlighted discrepancies between their beliefs and behaviour:

Reflecting on what I've usually done, when I speak out loud, you realise all the [behaviours] that are clearly problematic, and I wouldn't recommend someone else doing them. (P20)

Participants also found that having new routines depicted granularly and visually effectively communicated the ‘*instructions’* (P3) for agreed behaviour changes (‘*it’s like a recipe … it’s definitely easier to see [what I had to do when it's] on paper’*; P15).

Participants appreciated the simplicity of tackling poor sleep by attempting what they perceived to be minor, non-disruptive adjustments to existing routines, which were easy to adhere to:

It made me realise where I could take things away and where things could be changed … and still get all the key things done, but also give me that extra hour. (P10)

*Raised awareness of own sleeping patterns.* Sleep hygiene routines were typically ‘*not really something I had thought about’* (P14). Script elicitation increased awareness of the unhygienic and habitual nature of their behaviours, which inspired change:

It brought to my attention how I literally do that [evening routine] every night … [and] I actually do it in that specific order every night. I didn't quite realise how meticulous I was about it. (P15)

For many participants, understanding their evening routines gave them a greater sense of autonomy over their sleep:

I always thought I just sleep badly and that's like a trait I have. I didn't think there was too much I could do to really solve it. […] Looking at my routines and patterns in the evening definitely made me realise things I'm doing which aren't helping my situation. (P2)

*Facilitators and barriers to adherence to new scripts.* Many participants reported unanticipated barriers to adhering to their new scripts. These included psychological barriers such as momentary motivation dips, boredom, temptation, and a lack of self-discipline (‘*sometimes you don't want to stick to a routine, it just depends on your mood’;* P22). Others reported barriers outside of their control, including behaviour of others (e.g. family, housemates), or unanticipated stressors. These derailed performance of at least one behaviour within the new script, which precluded adherence to subsequent actions:

I couldn't turn all my electronics off because [my partner] was working … he finished work around [midnight] … it’s too late to do meditation. (P24)

Some participants reportedly adjusted their scripts to reduce the likelihood of being derailed by motivational or external problems. Some told friends and family about their new routine, both to invite social support and to publicly commit to their plan, making themselves accountable to others. Among those who successfully implemented their new routines, experiencing benefits spurred motivation to continue **(‘***seeing how much more energised I am … I definitely want to [continue to] follow [my new script]’;* P13).

For some, the habitual nature of existing routines acted as a powerful barrier to implementing changes (*‘I am too used to [eating at 8pm to] have dinner … at 7 instead of 8’;* P8). Conversely, those who were able to adhere to their planned routine reported forming habits and relying less on external aids (*‘[by] the end of the week it was just more like a routine for me, I didn't need to use the flow chart’;* P2).

*Experienced behavioural and health benefits.* Most participants reported successfully changing aspects of their routine. For most, the main modification involved reducing use of phones or tablets in bed, typically by leaving devices outside of the bedroom (Table 2). Successful behaviour change reportedly led many to experience improvements in sleep duration or quality (‘*I found that I wasn't waking up as much during the night’;* P16). Some participants expressed surprise at the magnitude and speed with which they experienced sleep benefits (‘*it worked remarkably, I thought it might take longer for it to have an effect’;* P19).

Better sleep reportedly translated into wellbeing benefits, including feeling ‘*more energised and more alert’* (P10) during the day, and improved mood (‘*I'm* *not as grumpy in the mornings … and more relaxed if something goes wrong*’; P1). Increased energy led some to experience enhanced social lives and greater productivity (‘*I was talking to my pals more. I was doing a lot more work, making sure it was done quicker’;* P15).

## Discussion

This study demonstrated feasibility and acceptability of a novel method for documenting and reconfiguring habitual evening sleep hygiene routines (‘script elicitation’). Quantitative data confirmed interest in the intervention among short-sleepers, zero attrition, and no detriments in sleep hygiene or quality following the intervention. Although participants pursued common sleep hygiene practices within their new routines, qualitative data showed that the intervention was deemed useful for raising awareness of behaviours and cues to which participants did not attend, so providing an informed basis for amending their routines. The acceptability of the intervention among our sample justifies progression to a controlled trial (Skivington et al., 2021).

Our script elicitation method involved an interviewer working with each participant to generate a detailed, personalised description of their typical evening, pre-sleep routines. Participants found this process enlightening, giving them new insight into their behaviours and empowering them to make changes. A lack of prior awareness of these behaviours supports previous assertions that sleep hygiene patterns are executed habitually, with minimal forethought (Rebar et al., 2020). Habitual actions are activated automatically due to learned associations between situational cues (such as preceding behaviours, e.g. ‘go into bedroom’) and responses to them (‘change into pyjamas’; Gardner, 2015). When people enact habitual behaviours, they can devote their finite mental resources to more demanding concurrent tasks, rather than to ongoing routine actions (Wood et al., 2002). This can mean that potentially problematic pre-sleep behaviours persist simply because people have little conscious oversight of them. It may appear contradictory to claim that participants had limited awareness of their behaviour prior to script elicitation, given that the elicitation technique required participants to self-report their actions. However, people tend to consciously represent well-practised behaviours as singular higher-order sequences (e.g. ‘getting changed’; Vallacher & Wegner, 1987), such that sub-components of these sequences (e.g. ‘remove clothes’, ‘get into pyjamas’) are executed outside of conscious awareness (Gardner et al., 2016). While people may not actively attend to fine-grained actions, they can voice procedural knowledge of enacting such behaviours, and doing so raises these behaviours into conscious awareness.

Elicitation of personalised scripts, and subsequent gains in knowledge and awareness, provided a basis for personally tailored intervention. While the quantitative data suggested no overall changes to sleep hygiene, in interviews participants reported generally adopting more hygienic evening sleep preparation behaviours and experiencing sleep and wellbeing improvements. Participants typically chose to remove or curtail common sleep-inhibiting behaviours (e.g. watching TV), and added common sleep-promotion actions (e.g. reading) without receiving sleep hygiene advice. This echoes research showing that people generally understand which behaviours affect sleep (Paterson et al., 2019), which suggests that educating people about the behavioural antecedents of good and bad sleep may not be optimal for promoting sleep hygiene (Murawski et al., 2018). Although our study was not designed to test effectiveness, results suggest that reflecting on and adapting habitual sleep preparation may be key to behaviour change. Interventions should support people in identifying *how* to change habitual sleep routines, rather than educating them on *which behaviours* to change.

Some participants struggled to adhere to new scripts, due to unanticipated external obstacles. This highlights two important limitations of script elicitation for sleep hygiene: it assumes both that evening routines are stable and consistent, and that people have autonomy over these routines. It is well-documented that situational changes, over which people have little control, can disrupt routines (Verplanken et al., 2018). Relatedly, when developing new scripts to follow, people may not adequately anticipate potential barriers. Future work might usefully refine the script elicitation method to focus either on aspects of sleep hygiene routines that are unlikely to be disrupted by unforeseen barriers, or to encourage greater anticipation and problem solving of barriers when developing new scripts.

Other limitations of script elicitation must be acknowledged. We allowed participants to identify behaviours to amend, which assumes awareness of sleep-conducive behaviours, and overlooks that some actions may impact more greatly on sleep hygiene. Script elicitation might be modified to fit an expert-led model of delivering behavioural guidance. Additionally, script elicitation requires intensive, one-to-one support. Although administration time was not recorded, script elicitation took up most of the baseline interview, which typically lasted over 35 mins. While a potentially useful addition to existing one-to-one sleep support interventions, future work might explore how to deliver script elicitation more efficiently (e.g. using automated ‘chatbot’ systems; Nadarzynski et al., 2019).

Our sampling strategy was limited. We used short sleep as a proxy for all sleep-related deficits, so recruited participants sleeping <6 h/night. Yet, sleep duration does not capture sleep quality or disturbance. While our participants reported enhanced sleep quality, future trials should evaluate the impact of script elicitation on a broad range of sleep outcomes. Additionally, we did not screen participants according to sleep hygiene. Above-midpoint baseline sleep hygiene scores suggest that we may have not trialled our intervention on the most relevant sample. Yet, participants described improving their sleep hygiene, implying that script elicitation might further enhance adequate sleep hygiene. Around half of our sample were students. University students tend to have poorer sleep than others (Lund et al., 2010), and are highly educated, so may be particularly health literate, and more likely to deem script elicitation acceptable. Similarly, our sample was predominantly female and White British, but women, and people of White ethnicities, tend to experience better sleep than other groups (Goel et al., 2005; Whinnery et al., 2014). Future studies should trial script elicitation among a broader demographic, with better representation of those most in need of sleep hygiene support.

Due to the ongoing Covid lockdown during recruitment, we conducted semi-structured interviews and administered script elicitation procedures to participants online, rather than in-person. This may have compromised the quality of interview data and intervention engagement (Johnson et al., 2021). Script elicitation procedures in previous studies have been conducted in-person (Greenland et al., 2013; Judah et al., 2013). However, the relative disadvantages of online compared to in-person interviewing are thought to arise from technological difficulties, reduced vocal clarity, or participants being distracted (Johnson et al., 2021). None of our participants reported technological difficulties nor were interpretation problems apparent. Additionally, the cognitively demanding nature of the script co-production task and the richness of the interview data obtained suggest that participants engaged deeply and meaningfully with the study procedures. While we would ideally have run interviews in-person, we do not view the use of online interviewing procedures as having notably diminished the acceptability of script elicitation among our participants.

Lastly, we cannot infer with certainty that experiences of online script elicitation procedures administered during a national lockdown would reflect experiences of delivery via alternative formats, or in non-lockdown conditions. However, interview data suggested that script elicitation was experienced positively because it raised participants’ awareness of their routines and inspired confidence in modifying these routines. These change mechanisms are not obviously tied to delivery format or setting. Although intervention experiences are necessarily context dependent (Skivington et al., 2021), our participants’ reflections likely capture experiences generalisable beyond the immediate context in which the script elicitation procedure was administered in this study.

Our findings suggested that a method for eliciting personalised information on evening sleep hygiene was feasible and acceptable to recipients. Participants’ receptiveness to and engagement with the script elicitation procedure indicated positive affective attitudes towards the intervention, and zero attrition rates suggested the intervention was not unduly burdensome. No detriments were observed in quantitative sleep hygiene or quality scores, suggesting the intervention was ethical, and participants reportedly experienced behavioural, sleep and wellbeing benefits, which suggests script elicitation has the potential to be effective. A rigorous controlled trial of the effectiveness of script elicitation versus treatment as usual (i.e. advice on which behaviours contribute to sleep hygiene; NHS, n.d.) for enhancing sleep hygiene and sleep is warranted.

## Supplementary Material

Supplemental Material

Supplemental Material

Supplemental Material

Supplemental Material

## Data Availability

Study data are available at https://osf.io/7963j/?view_only=9ec3667882f84e1cae56d463453c6e06.

## Note

1 Due to researcher error, the script elicitation segment of one interview was not audio-recorded so could not be coded for Table 2.

## Open Scholarship

This article has earned the Center for Open Science badges for Open Data and Open Materials through Open Practices Disclosure. The data and materials are openly accessible at https://osf.io/7963j/?view_only=9ec3667882f84e1cae56d463453c6e06 and https://osf.io/7963j/?view_only=9ec3667882f84e1cae56d463453c6e06.

## References

[CIT0001] Anderson, J. R. (1983). *The architecture of cognition*. Harvard University Press.

[CIT0002] Bargh, J. A. (1994). The four horsemen of automaticity: Intention, awareness, efficiency, and control as separate issues. In R. S. Wyer, & T. K. Srull (Eds.), *Handbook of social cognition* (pp. 1–40). Psychology Press.

[CIT0003] Blunden, S. L., Chapman, J., & Rigney, G. A. (2012). Are sleep education programs successful? The case for improved and consistent research efforts. *Sleep Medicine Reviews*, *16*(4), 355–370. 10.1016/j.smrv.2011.08.002 22104441

[CIT0004] Braun, V., & Clarke, V. (2019). Reflecting on reflexive thematic analysis. *Qualitative Research in Sport, Exercise and Health*, *11*(4), 589–597. 10.1080/2159676X.2019.1628806

[CIT0005] Braun, V., & Clarke, V. (2021). One size fits all? What counts as quality practice in (reflexive) thematic analysis? *Qualitative Research in Psychology*, *18*(3), 328–352. 10.1080/14780887.2020.1769238

[CIT0006] Centers for Disease Control and Prevention. (2016, July 15). *Sleep and sleep disorders: Tips for better sleep*. https://www.cdc.gov/sleep/about_sleep/sleep_hygiene.html

[CIT0007] Consensus Conference Panel, Watson, N. F., Badr, M. S., Belenky, G., Bliwise, D. L., Buxton, O. M., Buysse, D., Dinges, D. F., Gangwisch, J., Grandner, M. A., Kushida, C., Malhotra, R. K., Martin, J. L., Patel, S. R., Quan, S. F., & Tasali, E. (2015). Joint consensus statement of the American academy of sleep medicine and sleep research society on the recommended amount of sleep for a healthy adult: Methodology and discussion. *Sleep*, *38*(8), 1161–1183. 10.5665/sleep.4886 26194576 PMC4507722

[CIT0008] Corrente, M., & Bourgeault, I. (2022). Innovation in transcribing data: Meet otter.ai. *SAGE Research Methods: Doing Research Online*. 10.4135/9781529799033

[CIT0009] Exelmans, L., & Van den Bulck, J. (2021). “Glued to the tube”: The interplay between self-control, evening television viewing, and bedtime procrastination. *Communication Research*, *48*(4), 594–616. 10.1177/0093650216686877

[CIT0010] Gardner, B. (2015). A review and analysis of the use of ‘habit’ in understanding, predicting and influencing health-related behaviour. *Health Psychology Review*, *9*(3), 277–295. 10.1080/17437199.2013.876238 25207647 PMC4566897

[CIT0011] Gardner, B., Lally, P., & Rebar, A. L. (2020a). Does habit weaken the relationship between intention and behaviour? Revisiting the habit-intention interaction hypothesis. *Social and Personality Psychology Compass*, *14*(8), *e12553*. 10.1111/spc3.12553

[CIT0012] Gardner, B., Phillips, L., & Judah, G. (2016). Habitual instigation and habitual execution: Definition, measurement, and effects on behaviour frequency. *British Journal of Health Psychology*, *21*(3), 613–630. 10.1111/bjhp.12189 26991427

[CIT0013] Gardner, B., Rebar, A. L., & Lally, P. (2020b). Habit interventions. In M. S. Hagger, L. D. Cameron, K. Hamilton, N. Hankonen, & T. Lintunen (Eds.), *The handbook of behavior change* (pp. 599–616). Cambridge University Press.

[CIT0014] Goel, N., Kim, H., & Lao, R. P. (2005). Gender differences in polysomnographic sleep in young healthy sleepers. *Chronobiology International*, *22*(5), 905–915. 10.1080/07420520500263235 16298775

[CIT0015] Greenland, K., Iradati, E., Ati, A., Maskoen, Y. Y., & Aunger, R. (2013). The context and practice of handwashing among new mothers in Serang, Indonesia: A formative research study. *BMC Public Health*, *13*(1), 830. 10.1186/1471-2458-13-830 24020804 PMC3847175

[CIT0016] Hollands, G. J., Marteau, T. M., & Fletcher, P. C. (2016). Non-conscious processes in changing health-related behaviour: A conceptual analysis and framework. *Health Psychology Review*, *10*(4), 381–394. 10.1080/17437199.2015.1138093 26745243 PMC5214381

[CIT0017] Itani, O., Jike, M., Watanabe, N., & Kaneita, Y. (2017). Short sleep duration and health outcomes: A systematic review, meta-analysis, and meta-regression. *Sleep Medicine*, *32*, 246–256. 10.1016/j.sleep.2016.08.006 27743803

[CIT0018] Johnson, D. R., Scheitle, C. P., & Ecklund, E. H. (2021). Beyond the in-person interview? How interview quality varies across in-person, telephone, and skype interviews. *Social Science Computer Review*, *39*(6), 1142–1158. 10.1177/0894439319893612

[CIT0019] Judah, G., Gardner, B., & Aunger, R. (2013). Forming a flossing habit: An exploratory study of the psychological determinants of habit formation. *British Journal of Health Psychology*, *18*(2), 338–353. 10.1111/j.2044-8287.2012.02086.x 22989272

[CIT0020] Lally, P., & Gardner, B. (2013). Promoting habit formation. *Health Psychology Review*, *7*(Suppl 1), S137–S158. 10.1080/17437199.2011.603640

[CIT0021] Lancaster, G. A., Dodd, S., & Williamson, P. R. (2004). Design and analysis of pilot studies: Recommendations for good practice. *Journal of Evaluation in Clinical Practice*, *10*(2), 307–312. 10.1111/j..2002.384.doc.x 15189396

[CIT0022] Levenson, J. C., Shensa, A., Sidani, J. E., Colditz, J. B., & Primack, B. A. (2016). The association between social media use and sleep disturbance among young adults. *Preventive Medicine*, *85*, 36–41. 10.1016/j.ypmed.2016.01.001 26791323 PMC4857587

[CIT0023] Lund, H. G., Reider, B. D., Whiting, A. B., & Prichard, J. R. (2010). Sleep patterns and predictors of disturbed sleep in a large population of college students. *Journal of Adolescent Health*, *46*, 124–132. 10.1016/j.jadohealth.2009.06.016 20113918

[CIT0024] Mastin, D. F., Bryson, J., & Corwyn, R. (2006). Assessment of sleep hygiene using the sleep hygiene index. *Journal of Behavioral Medicine*, *29*(3), 223–227. 10.1007/s10865-006-9047-6 16557353

[CIT0025] Mead, M. P., & Irish, L. A. (2022). The theory of planned behaviour and sleep opportunity: An ecological momentary assessment. *Journal of Sleep Research*, *31*(1), e13420. 10.1111/jsr.13420 34137110 PMC8674382

[CIT0026] Michie, S., Richardson, M., Johnston, M., Abraham, C., Francis, J., Hardeman, W., Eccles, M. P., Cane, J., & Wood, C. E. (2013). The behavior change technique taxonomy (v1) of 93 hierarchically clustered techniques: Building an international consensus for the reporting of behavior change interventions. *Annals of Behavioral Medicine*, *46*(1), 81–95. 10.1007/s12160-013-9486-6 23512568

[CIT0027] Murawski, B., Wade, L., Plotnikoff, R. C., Lubans, D. R., & Duncan, M. J. (2018). A systematic review and meta-analysis of cognitive and behavioral interventions to improve sleep health in adults without sleep disorders. *Sleep Medicine Reviews*, *40*, 160–169. 10.1016/j.smrv.2017.12.003 29397329

[CIT0028] Nadarzynski, T., Miles, O., Cowie, A., & Ridge, D. (2019). Acceptability of artificial intelligence (AI)-led chatbot services in healthcare: A mixed-methods study. *Digital Health*, *5*, 1–12. 10.1177/2055207619871808 PMC6704417 31467682

[CIT0029] NHS. (n.d.). *Top tips to get to sleep better*. https://www.nhs.uk/every-mind-matters/mental-health-issues/sleep/#top-tips

[CIT0030] Oh, J. H., Yoo, H., Park, H. K., & Do, Y. R. (2015). Analysis of circadian properties and healthy levels of blue light from smartphones at night. *Scientific Reports*, *5*(1), 11325. 10.1038/srep11325 26085126 PMC4471664

[CIT0031] Parrott, A. C., & Hindmarch, I. (1980). The Leeds sleep evaluation questionnaire in psychopharmacological investigations—A review. *Psychopharmacology*, *71*(2), 173–179. 10.1007/BF00434408 6777817

[CIT0032] Paterson, J. L., Reynolds, A. C., Duncan, M., Vandelanotte, C., & Ferguson, S. A. (2019). Barriers and enablers to modifying sleep behavior in adolescents and young adults: A qualitative investigation. *Behavioral Sleep Medicine*, *17*(1), 1–11. 10.1080/15402002.2016.1266489 28067547

[CIT0033] Peltz, J. S., & Rogge, R. D. (2016). The indirect effects of sleep hygiene and environmental factors on depressive symptoms in college students. *Sleep Health*, *2*(2), 159–166. 10.1016/j.sleh.2016.01.007 28923260

[CIT0034] Rebar, A. L., Reynolds, A. C., Ferguson, S. A., & Gardner, B. (2020). Accounting for automatic processes in sleep health. *Journal of Sleep Research*, *29*(5), e12987. 10.1111/jsr.12987 32166798

[CIT0035] Sekhon, M., Cartwright, M., & Francis, J. J. (2017). Acceptability of healthcare interventions: An overview of reviews and development of a theoretical framework. *BMC Health Services Research*, *17*(1), 88. 10.1186/s12913-017-2031-8 28126032 PMC5267473

[CIT0036] Skivington, K., Matthews, L., Simpson, S. A., Craig, P., Baird, J., Blazeby, J. M., Boyd, K. A., Craig, N., French, D. P., McIntosh, E., Petticrew, M., Rycroft-Malone, J., White, M., & Moore, L. (2021). A new framework for developing and evaluating complex interventions: Update of medical research council guidance. *BMJ*, *374*, n2061. 10.1136/bmj.n2061 34593508 PMC8482308

[CIT0037] Sleep Foundation. (2022). *How to Sleep Better*. Sleep Foundation. https://www.sleepfoundation.org/sleep-hygiene/healthy-sleep-tips

[CIT0038] The Sleep Council. (2017). *The Great British Bedtime Report 2017*.

[CIT0039] Vallacher, R. R., & Wegner, D. M. (1987). What do people think they’re doing? Action identification and human behavior. *Psychological Review*, *94*(1), 3–15. 10.1037/0033-295X.94.1.3

[CIT0040] Verplanken, B., Roy, D., & Whitmarsh, L. (2018). Cracks in the wall: Habit discontinuities as vehicles for behaviour change. In B. Verplanken (Ed.), *The psychology of habit* (pp. 189–206). Springer.

[CIT0041] Watanabe, M., Kikuchi, H., Tanaka, K., & Takahashi, M. (2010). Association of short sleep duration with weight gain and obesity at 1-year follow-up: A large-scale prospective study. *Sleep*, *33*(2), 161–167. 10.1093/sleep/33.2.161 20175399 PMC2817903

[CIT0042] We Are Social. (2022). *Digital 2022: The United Kingdom*. https://datareportal.com/reports/digital-2022-united-kingdom

[CIT0043] Whinnery, J., Jackson, N., Rattanaumpawan, P., & Grandner, M. A. (2014). Short and long sleep duration associated with race/ethnicity, sociodemographics, and socioeconomic position. *Sleep*, *37*(3), 601–611. 10.5665/sleep.3508 24587584 PMC3920327

[CIT0044] Wood, W., Quinn, J. M., & Kashy, D. A. (2002). Habits in everyday life: Thought, emotion, and action. *Journal of Personality and Social Psychology*, *83*(6), 1281–1297. 10.1037/0022-3514.83.6.1281 12500811

[CIT0045] YouGov. (2022). *How many hours Brits sleep at night*. https://yougov.co.uk/topics/health/trackers/how-many-hours-brits-sleep-at-night

