# Supplementary material for: Feasibility and acceptability of a personalised script-elicitation method for improving evening sleep hygiene habits
Source: Health Psychol Behav Med. 2023 Jan 1;11(1):2162904. doi: 10.1080/21642850.2022.2162904 (PMC9815428; doi:10.1080/21642850.2022.2162904)
Supplement: Supplemental Material [file RHPB_A_2162904_SM4037.docx]

| *Script elicitation component* | *Script elicitation stage* | *Potential behaviour change techniques** |
| --- | --- | --- |
| Identification of cues and preceding behaviours in existing script | 1. Eliciting existing script | - Information about antecedents |
| Interviewer supporting participant in identification of existing script, and in modification of script | 1. Eliciting existing script  2. Creating alternative script | - Social support (practical) |
| Identification of which behaviour(s) to add, remove, or reorganise within the script | 2. Creating alternative script | - Goal-setting (behaviour)  - Problem solving |
| Identification of which behaviour(s) to add to the script | 2. Creating alternative script | - Action planning |
| Identification of cues to new behaviours within script | 2. Creating alternative script | - Prompts/cues |
| Identification of behaviours or cues to remove from script, as means of reducing likelihood of a subsequent behaviour | 2. Creating alternative script | - Reduce prompts/cues  - Restructuring the physical environment  - Restructuring the social environment  - Avoidance/reducing exposure to cues for the behaviour |
| Identification of new, wanted behaviour to repeatedly perform at given site within the script | 2. Creating alternative script | - Habit formation |
| Identification of alternative behaviours to perform in place of unwanted, habitual behaviours | 2. Creating alternative script | - Behaviour substitution  - Habit reversal |
| Envisioning performance of new script | 2. Creating alternative script | - Mental rehearsal of successful performance |

* Behaviour change techniques derived from the BCT Taxonomy v1 (Michie et al., 2013). Script elicitation is a personally-tailored intervention technique, such that not all script elicitation components will be used by all participants, nor will all potential behaviour change techniques necessarily be deployed.
