# Supplementary material for: Feasibility and acceptability of a personalised script-elicitation method for improving evening sleep hygiene habits
Source: Health Psychol Behav Med. 2023 Jan 1;11(1):2162904. doi: 10.1080/21642850.2022.2162904 (PMC9815428; doi:10.1080/21642850.2022.2162904)
Supplement: Supplemental Material [file RHPB_A_2162904_SM4034.docx]

**Manuscript:** *Feasibility and acceptability of a personalised script-elicitation method for improving evening sleep hygiene habits*

**Consolidated criteria for reporting qualitative studies (COREQ): 32-item checklist**

Developed from:

Tong A, Sainsbury P, Craig J. Consolidated criteria for reporting qualitative research (COREQ): a 32-item checklist for interviews and focus groups. *International Journal for Quality in Health Care*. 2007. Volume 19, Number 6: pp. 349 – 357

| **No. Item** | **Guide questions/description** | **Relevant excerpt** |
| --- | --- | --- |
| **Domain 1: Research team and reﬂexivity** |  |  |
| *Personal Characteristics* |  |  |
| 1. Inter viewer/facilitator | Which author/s conducted the inter view or focus group? | “Interviews were undertaken by one of two female postgraduate (Masters) Health Psychology students (AA, BB).” |
| 2. Credentials | What were the researcher’s credentials? E.g. PhD, MD | “Interviews were undertaken by one of two female postgraduate (Masters) Health Psychology students (AA, BB).” |
| 3. Occupation | What was their occupation at the time of the study? | Masters Health Psychology students |
| 4. Gender | Was the researcher male or female? | Female |
| 5. Experience and training | What experience or training did the researcher have? | “Neither had conducted real-world research interviews previously. Both were trained in research interviewing by CC and script elicitation administration by DD.” |
| *Relationship with participants* |  |  |
| 6. Relationship established | Was a relationship established prior to study commencement? | “Aside from email correspondence to arrange the interview, no relationship was established with any participant prior to interview, nor did participants have any knowledge about the interviewer other than that they were a psychology researcher who wished to test a method for improving sleep hygiene..” |
| 7. Participant knowledge of the interviewer | What did the participants know about the researcher? e.g. personal goals, reasons for doing the research | “Aside from email correspondence to arrange the interview, no relationship was established with any participant prior to interview, nor did participants have any knowledge about the interviewer other than that they were a psychology researcher who wished to test a method for improving sleep hygiene.” |
| 8. Interviewer characteristics | What characteristics were reported about the inter viewer/facilitator? e.g. Bias, assumptions, reasons and interests in the research topic | “Aside from email correspondence to arrange the interview, no relationship was established with any participant prior to interview, nor did participants have any knowledge about the interviewer other than that they were a psychology researcher who wished to test a method for improving sleep hygiene.” |
| **Domain 2: study design** |  |  |
| *Theoretical framework* |  |  |
| 9. Methodological orientation and Theory | What methodological orientation was stated to underpin the study? e.g. grounded theory, discourse analysis, ethnography, phenomenology, content analysis | “‘codebook’ form of inductive Thematic Analysis” |
| *Participant selection* |  |  |
| 10. Sampling | How were participants selected? e.g. purposive, convenience, consecutive, snowball | “Participants were recruited in June 2020 using convenience sampling methods, via online advertisements on social media (LinkedIn, Facebook, Twitter) and an advert in an all-staff circular email at an inner-city UK university.” |
| 11. Method of approach | How were participants approached? e.g. face-to-face, telephone, mail, email | “…via online advertisements on social media (LinkedIn, Facebook, Twitter) and an advert in an all-staff circular email at an inner-city UK university.”  “[Participants] provided their interview availability, and email address to receive interview confirmation […] email correspondence to arrange the interview” |
| 12. Sample size | How many participants were in the study? | “Twenty-four participants completed the study” |
| 13. Non-participation | How many people refused to participate or dropped out? Reasons? | “Those who came forward after 24 participants had signed up were added to a reserve list, but nobody dropped out, so the reserve list was not used.”  “All 24 participants completed follow-up (0% attrition)” |
| *Setting* |  |  |
| 14. Setting of data collection | Where was the data collected? e.g. home, clinic, workplace | “Participants … completed an online interview via Microsoft Teams” |
| 15. Presence of non-participants | Was anyone else present besides the participants and researchers? | “Only the interviewer and participant were present in each interview.” |
| 16. Description of sample | What are the important characteristics of the sample? e.g. demographic data, date | “Twenty-four participants completed the study (19 females, 5 males; age 18-66 years [Mean=29.3y, SD=13.2y]). Participants reported sleeping on average 5.4hrs/night on weekdays, and 5.5hrs at weekends. They were most commonly White British (58%), full-time students (54%), and living with their parents (42%; see Table 1).”  See also Table 1 |
| *Data collection* |  |  |
| 17. Interview guide | Were questions, prompts, guides provided by the authors? Was it pilot tested? | “Both interview schedules were piloted by AA and BB in mock interviews with friends.”  Interview schedules provided as supplementary material |
| 18. Repeat interviews | Were repeat inter views carried out? If yes, how many? | Two interviews were carried out.  “Participants … completed an online interview […] one week later, … they also gave a second, semi-structured interview” |
| 19. Audio/visual recording | Did the research use audio or visual recording to collect the data? | “Both interviews were audio-recorded” |
| 20. Field notes | Were ﬁeld notes made during and/or after the inter view or focus group? | No |
| 21. Duration | What was the duration of the inter views or focus group? | “[The first] interviews lasted between 17-59mins (mean 36min, SD=10). […] The second interviews lasted between 2-23mins (mean 8min, *SD*=4).” |
| 22. Data saturation | Was data saturation discussed? | Saturation for data collection purposes: N/A  (“budgetary limits … imposed a maximum sample of 24 participants”)  Saturation for data analysis purposes:  “Analysis continued until AA and CC agreed that no meaningful additional themes or codes were emerging.” |
| 23. Transcripts returned | Were transcripts returned to participants for comment and/or correction? | No |
| **Domain 3: analysis and ﬁndings** |  |  |
| *Data analysis* |  |  |
| 24. Number of data coders | How many data coders coded the data? | Three  “AA, BB and CC first independently familiarised themselves with and subsequently coded three transcripts, assigning labels to pertinent events within the data. Next, the three coders met to agree a preliminary, inductively derived structure of codes and clusters of codes (i.e., themes), to guide subsequent analysis. Next, this structure was applied and iteratively refined by AA to code all remaining data. AA met regularly with CC to review themes, assign appropriate theme labels, and confirm credibility of interpretations. Analysis continued until AA and CC agreed that no meaningful additional themes or codes were emerging.” |
| 25. Description of the coding tree | Did authors provide a description of the coding tree? | No – this is available on request |
| 26. Derivation of themes | Were themes identiﬁed in advance or derived from the data? | Derived from the data  “AA, BB and CC first independently familiarised themselves with and subsequently coded three transcripts, assigning labels to pertinent events within the data. Next, the three coders met to agree a preliminary, inductively derived structure of codes and clusters of codes (i.e., themes), to guide subsequent analysis. Next, this structure was applied and iteratively refined by AA to code all remaining data. AA met regularly with CC to review themes, assign appropriate theme labels, and confirm credibility of interpretations. Analysis continued until AA and CC agreed that no meaningful additional themes or codes were emerging.” |
| 27. Software | What software, if applicable, was used to manage the data? | Not applicable – no software was used to manage the data |
| 28. Participant checking | Did participants provide feedback on the ﬁndings? | No |
| *Reporting* |  |  |
| 29. Quotations presented | Were participant quotations presented to illustrate the themes/ﬁndings? Was each quotation identiﬁed? e.g. participant number | Yes – throughout ‘Qualitative data’ subsection of Results |
| 30. Data and ﬁndings consistent | Was there consistency between the data presented and the ﬁndings? | Yes |
| 31. Clarity of major themes | Were major themes clearly presented in the ﬁndings? | Yes |
| 32. Clarity of minor themes | Is there a description of diverse cases or discussion of minor themes? | Not applicable |
