# Supplementary material for: Feasibility and acceptability of a personalised script-elicitation method for improving evening sleep hygiene habits
Source: Health Psychol Behav Med. 2023 Jan 1;11(1):2162904. doi: 10.1080/21642850.2022.2162904 (PMC9815428; doi:10.1080/21642850.2022.2162904)
Supplement: Supplemental Material [file RHPB_A_2162904_SM4000.docx]

**Baseline interview**

** Data from these sections were not analysed in the present study*

**Existing sleep patterns***

Your answers to our questionnaire indicate that you typically sleep for less than 6 hours per night. What does sleep mean to you, and can you tell me your experience with it?

Can you tell me a bit more about your sleep patterns?

*Possible prompts:*

- - How well do you sleep?
  - What time do you go to bed?
  - What time do you usually get up?
  - How varied is your sleep routine?
  - Do you wake up often in the night?
  - If you do wake up during the night, are you able to go back to sleep easily?

Have you always slept for short periods of time? (If not, how long has your routine been like this?)

- *Possible prompts:*
  - Are you aware of anything that caused you to start sleeping for short(er) periods?

Why do you think you sleep for short periods of time?

- *Possible prompt:*
  - What, if anything, stops you from sleeping for longer periods of time?

How (if at all) does your short sleep affect you during the daytime?

- *Possible prompts:*
  - Do you feel tired during the day?
  - Does it affect your work performance?

Has your sleep been affected by the Covid-19 pandemic? If so, how?

**Attempts to change sleep patterns***

Have you ever tried to change your sleeping patterns so that you get more sleep? (If so, what did you try, and how effective was it?)

- If it was effective, what led you to start sleeping for short periods again?

Imagine that someone with the exact same quality of sleep, and duration of sleep, as you asked you to help them to improve their sleep. What would you recommend to them?

- *Possible follow-up:* What challenges do you think you would face in improving their sleep?

[*Script elicitation procedure here]*

**Reflections on script elicitation procedure**

How did you find seeing your routine displayed liked that?

- *Possible follow-up:* Do you think it was helpful? Why/why not?

**Follow up interview**

**Reflections on attempts to adhere**

How did you get on with trying to change your sleep routine?

- *Possible prompt:* Were you able to make any changes to your routine?

Did you notice any changes to your sleep?

Did you make any other changes relating to your sleep behaviours?

**Further reflections on script elicitation procedure**

How useful was the plan that we drew up last week?

Did you use the flowchart that we gave you?

Did you come across any barriers to making the changes you planned to make?

- If so, did you manage to overcome them?
  - If yes, how?
  - If no, why not?

How helpful in general was the first interview to you?

- *Possible prompts:*
  - What, if anything, did you take from the first interview? (i.e. did you learn anything new?)
- Any specific aspects of the first interview?

Would you recommend taking part to someone with the same sleeping patterns as you?

That is all I wanted to ask you today. Is there anything else you wanted to ask me?
