# Supplementary material for: Feasibility and acceptability of a personalised script-elicitation method for improving evening sleep hygiene habits
Source: Health Psychol Behav Med. 2023 Jan 1;11(1):2162904. doi: 10.1080/21642850.2022.2162904 (PMC9815428; doi:10.1080/21642850.2022.2162904)
Supplement: Supplemental Material [file RHPB_A_2162904_SM3585.docx]

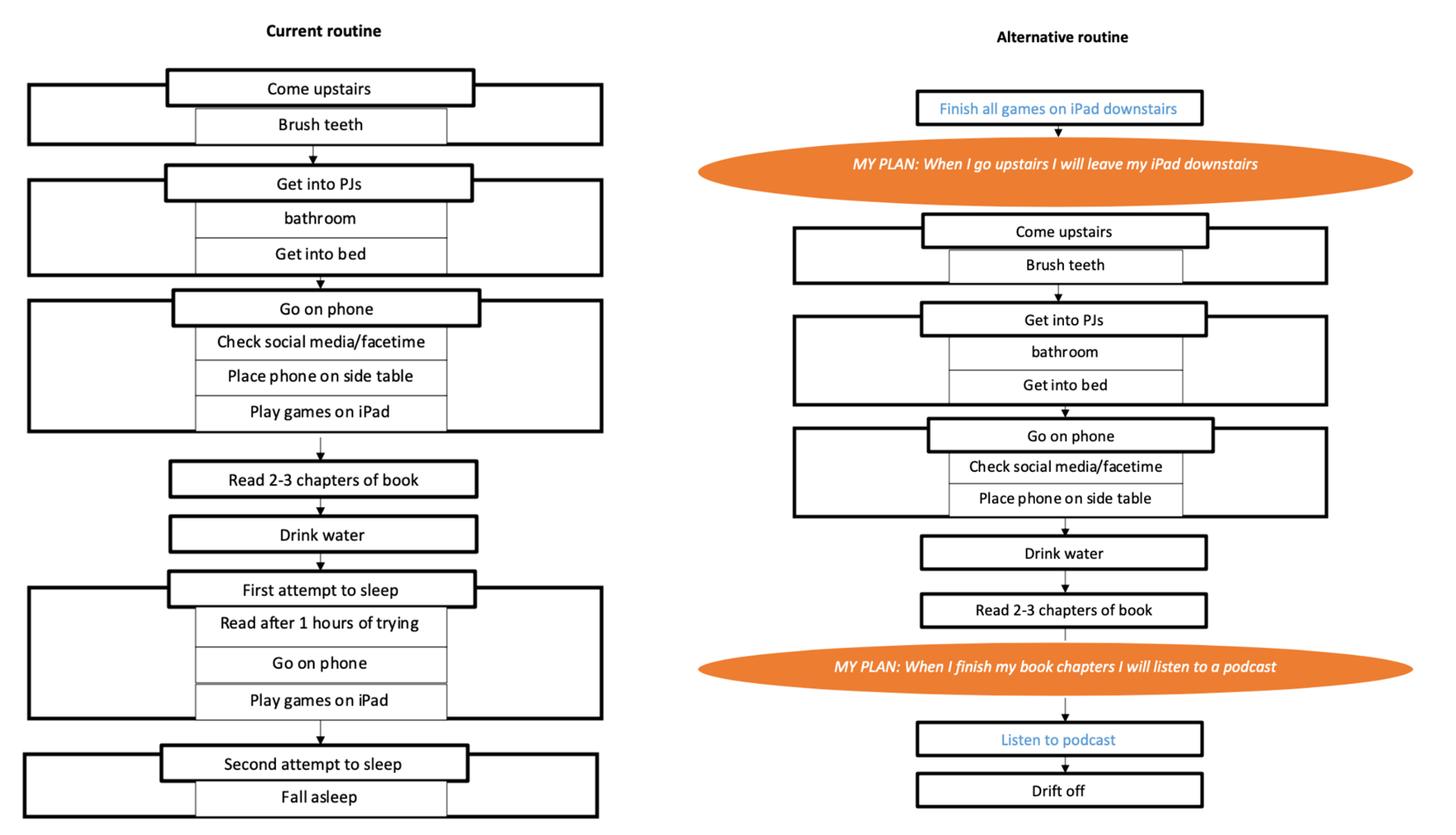


NB: In Alternative Routine, blue text denotes a behaviour added to the script, and orange ovals describe action plans to facilitate adherence to these behaviours
